# In Whipple's procedure, which anastomotic technique has lower leak rate; Pancreaticogastostomy or Pancreatojejunostomy?

**DOI:** 10.1016/j.amsu.2020.12.042

**Published:** 2020-12-30

**Authors:** Rashid Ibrahim, Sabry Abounozha, Hossam Nawara, Awad Alawad

**Affiliations:** a University Hospitals Plymouth NHS Trust, Plymouth, UK; b Northumbria Healthcare NHS Foundation Trust, Northumbria, UK; c University Hospital of Wales, Cardiff, UK

**Keywords:** Anastomotic, Leak, Pancreaticogastostomy, Pancreatojejunostomy, Whipple's

## Abstract

A best evidence topic has been constructed using a described protocol. The three-part question addressed was: in patient with Whipple's procedure which anastomotic technique has lower leak rate pancreaticogastostomy (PG) or pancreatojejunostomy (PJ)? Using the reported search, 38 articles were found; out of this six studies were deemed to be suitable to answer the question. The outcomes assessed were incidence of anastomotic leaks (pancreatic fistula) in both techniques PG and PJ. In conclusion, the best evidence showed that PG anastomosis has lower incidence of pancreatic fistula in comparison to PJ anastomosis.

## Highlights

• Aim: Comparison of pancreatic fistula rate among pancreaticogastostomy (PG) vs pancreatojejunostomy (PJ) anastomosis.
• Result: Six randomized controlled trials were included.
• Conclusion: The best evidence showed that PG anastomosis has lower incidence of pancreatic fistula than PJ anastomosis.

## 1 Introduction

This BET was designed using a framework outlined by the International Journal of Surgery [1]. This format was used because a preliminary literature search suggested that the available evidence is of insufficient quality to perform a meaningful meta-analysis. Pancreatic anastomosis is regarded by most surgeons as one of the most challenging steps of Whipple's procedure. Pancreatic anastomotic leakage is one of the main causes of post-operative morbidity and mortality. The aim from this article is to review which technique has the lower anastomotic leak rate Pancreaticogastostomy vs Pancreatojejunostomy? The unique part of this article is that it provides an evidence-based answers to the above mentioned clinical questions, using a systematic approach of reviewing the literature.

## 2 Clinical scenario

A senior surgical trainee is assisting in a difficult Whipple's procedure, the consultant is about to perform the pancreatic anastomosis, the trainee is wondering would it be better to perform PG or PJ in order to reduce the incidence of pancreatic leak?

## 3 Three-part question

[In patient with Whipple procedure] which anastomotic technique [has a lower leak rate] [PG or PJ]?

## 4 Search strategy

A. Embase 1974 to October 2020 using the OVID interface:

[Whippel's procedure OR Pancreaticoduodenectomy]AND [leak OR leaks OR fistula] AND [pancreaticogastostomy OR PG] AND [pancreatojejunostomy OR PJ]

B. Medline using the PubMed interface:

[Whippel's procedure OR Pancreaticoduodenectomy]AND [leak OR leaks OR fistula] AND [pancreaticogastostomy OR PG] AND [pancreatojejunostomy OR PJ].

The results were limited to English articles and human studies.

**Inclusion criteria**: all original articles that review the incidence anastomotic leak among patients who underwent Pancreaticogastostomy and Pancreatojejunostomy.

**Exclusion criteria**: case reports, systematic reviews, letters to the editor, conference abstracts.

## 5 Search outcome

A total of 38 papers were found using both search engines. Out of these 28 papers were excluded because they were irrelevant based on the titles and or the abstracts. Ten full-text articles were screened and assessed for eligibility. From these, six papers were identified to provide the best evidence to answer the question. The definition of pancreatic fistula used on the article based on the International Study Group for pancreatic fistula definition (ISGPF) [2].

## 6 Result: see the Table 1

**Table 1:** Search result

Author, date of publication, journal and country	Study type and level of evidence	Patient group and Follow up	Outcomes	Key results	Additional comments
Topal et al. 2013 Lancet Oncol Belgium	Multi centre Randomized controlled trial level II	Total of 329 whippel's procedure Group 1: PG = 162 Group2: PJ = 167 Follow up = 2 months	The primary endpoint was postoperative pancreatic fistula	Group1 = 13 (8·0%) Group2 = 33 (19·8%) p = 0·002 Statically significant low anastomotic leak in PG	-multicentre centre, -large sample size, -preopertive randamiation -Objective definition of pancreatic fistula
Duffas et al. 2004 American Journal of Surgery France	Multi centre Randomized controlled trial level II	Total 149 were randomized Group 1: PG = 81 Group2:PJ = 68 Follow up = not mentioned	The primary endpoint was postoperative pancreatic fistula	Group1 = 13(16%) Group2 = 14 (20%) not statistically significant difference	-multi centre -relatively small sample size -no exact definition of pancreatic fistula used –
Wellner et al. 2012 JGastrointest Surg Germany	Randomized controlled trial level II	Total 116 were randomized Group 1: PG = 59 Group2:PJ = 57 Follow up = not mentioned	The primary endpoint was postoperative pancreatic fistula	Group1 = 10% Group2 = 12% p = 0.775 not statistically significant difference	-Single centre -relatively small sample size -intraoperative randomization. –
Figueras, et al. 2013 British Journal of Surgery Spain	Randomized Controlled Trial Level II	123 patients randomized, underwent PJ and Group 1: PG = 65 Group2:PJ = 58 Follow up = 60 days	The primary endpoint was postoperative pancreatic fistula	Group1 = 10 (15%) Group2 = 20 (34%) P = 0·014 statistically significant difference	Single centre -relatively small sample size -no subgroup analysis based on duct diameter or specific pathology was performed
Nakeeb et al. 2013 HPB Egypt	Randomized controlled trial level II	Total 90 patients Randomized into: Group 1: PG = 45 Group2:PJ = 45 pancreatic fistula is defined as increased levels of amylase in the effluent drain three times higher than the plasma levels after postoperative day 3. Follow up = 1 year	The primary endpoint was postoperative pancreatic fistula	Group1 = 10 (22%) Group2 = 9 (20%) P = 0.796 not statistically significant difference	-Single centre, -Small sample size no subgroup analysis based on duct diameter or specific pathology was performed operations were performed by eight surgeons, which may have represented a source of bias
Takano et al. British Journal of Surgery 2000 Japan	Randomized controlled trial level II	Total 90 patients Randomized into: Group 1: PG = 73 Group2:PJ = 69 Follow up = not mentioned	The primary endpoint was postoperative pancreatic fistula	Group1 = (0%) Group2 = (13%) P = 0.014 statistically significant difference	-Single centre, -Small sample - each procedure was perform in different centre with different team

## 7 Discussion

Pancreatic anastomotic leakage is one of the main causes of morbidity and mortality after Whipple's procedure [3]. The incidence of pancreatic fistula (PF) varies greatly in different reports due to the different definitions of fistula [4]. In our review most of the articles adopt the International Study Group for pancreatic fistula (ISGPF) definition [2]. Many techniques have been described for joining the pancreatic stump either with the jejunum or with the stomach, with or without internal or external drainage of the pancreatic duct [5]. However, there are still some conflicting results in the literature regarding which technique has the lower leak rate. In this review we have compared six of the largest randomized controlled trials that compare the incidence of pancreatic fistula among the patients who had PG and PJ.

In our review, Three randomized control trials showed no statically significant difference in the rate of pancreatic fistulas among the two techniques these were conducted by Nakeeb et [6], Duffas et al. [7], and Wellner et al. [8]. However, these trials have some limitations such as small sample size, lack of preoperative randomization and absence of subgroup analysis based on pancreatic duct diameter.

In the year 2000, Takano et al. [9] published an RCT in the British Journal of Surgery which showed that PG has significantly low incidence of pancreatic fistula in comparison to PJ. However the main limitation of this review is the small sample size. In 2013, Figueras et al. [10] published a relatively larger size single centre RCT, which also has the same conclusion that PG is superior to PJ with regards to the pancreatic fistula. Topal et al. [11] in the same year published the largest multicentre RCT; it was the only multicentre study to use a stratified design to assess the outcome of PG compared to PJ after. The result proved that PG has statistically significant lower rate of pancreatic fistula in comparison to PJ.

### 7.1 Clinical bottom line

According to the above articles, the best evidence showed that PG anastomosis is associated with lower rate of pancreatic fistula in comparison to PJ anastomosis. The recommendation of the authors is that PG is better than PJ, particularly with regard to the incidence of pancreatic fistula.

### 7.2 Limitation of this review

Most of the articles except two have small sample size and are single centre, also in most article there is lack of preoperative randomization and absence of subgroup analysis based on pancreatic duct diameter. Most articles didn't mention the period of post-operative follow up.

In order to overcome these limitations, the authors do recommend a well design, large multicentre randomized control trials with long period of follow up.

## Ethical approval

Not applicable.

## Sources of funding

None.

## Author contribution

RI: conducted the literature search and wrote the paper.

SA: assisted in the literature search and Writing of paper.

HN: assisted in writing of paper.

AA: assisted in the literature search, editing of writing.

## Provenance and peer review

Not commissioned, externally peer-reviewed.

## Declaration of competing interest

None.
